# REVIVE: a computational platform for systematically identifying rejuvenating chemical and genetic perturbations

**DOI:** 10.18632/aging.206342

**Published:** 2025-11-25

**Authors:** Sascha Jung, Javier Arcos Hodar, Tejwasi Venkata S. Badam, Antonio del Sol

**Affiliations:** 1 Computational Biology Group, CIC bioGUNE-BRTA (Basque Research and Technology Alliance), Bizkaia Technology Park, Derio 48160, Spain; 2 Department of Biochemistry and Molecular Biology, University of the Basque Country, UPV/EHU, Leioa, Spain; 3 Computational Biology Group, Luxembourg Centre for Systems Biomedicine (LCSB), University of Luxembourg, Esch-sur-Alzette L-4362, Luxembourg; 4 Ikerbasque, Basque Foundation for Science, Bilbao, Bizkaia 48012, Spain

**Keywords:** computational modeling, machine learning, aging, rejuvenation, drug repurposing

## Abstract

Great efforts have been devoted to discovering rejuvenation strategies that counteract age-related functional decline and improve cellular functions in humans. However, new discoveries are currently driven by expert knowledge and require large amounts of resources. Here, we present REVIVE (Rejuvenation Estimation Via Insightful Virtual Experiments), the first computational framework for systematically predicting chemical and genetic perturbations that can restore a youthful transcriptional state based on gene expression data. REVIVE leverages age predictions to detect significant rejuvenating effects and quantifies the impact of perturbations on the hallmarks of aging. When applied to a large-scale *in silico* screen of more than 10000 compounds and genetic perturbations, REVIVE recapitulates known interventions as well as 477 novel compounds that restore a more youthful transcriptional state improving multiple aging hallmarks. Finally, we demonstrate the utility of REVIVE for repurposing perturbations to revert aged transcriptional states.

## INTRODUCTION

Aging is a heterogeneous process that manifests in molecular and cellular aberrations leading to impaired tissue functioning. In this regard, the aging phenotype has been characterized by a set of hallmarks responsible for the functional decline, including cellular senescence, stem cell exhaustion and genomic instability [1, 2]. Although first evidence has been presented that the aging process is malleable almost a century ago, it has long been considered to be irreversible [3]. However, several intervention strategies have been discovered in recent years that can partially revert the hallmarks of aging, such as heterochronic parabiosis, rapamycin, senolytics and partial reprogramming [4]. In spite of the fact that these strategies have been shown to be effective in preclinical animal models, their translation to humans remains a challenge. For instance, heterochronic parabiosis, in which the blood streams of young and old individuals are connected, counteracts the aging phenotype across several tissues of an organism but is not translatable to the clinics. In addition, significant safety concerns have been raised in the context of senolytics and partial reprogramming. While the accumulation of senescent cells with age can induce inflammation, it can also exert beneficial effects on other cells such as the secretion of growth factors [5]. Thus, the complete and untargeted inhibition of senescent cells is considered to be detrimental. Moreover, partial reprogramming bears the risk of neoplasm formation due to the dedifferentiation of cells and their concomitant loss of cell identity [6]. Hence, there is a need for alternative rejuvenation interventions that are safe, effective and translatable to the clinics.

The discovery of rejuvenating interventions has traditionally been driven by experimental efforts that are laborious and require vast amounts of resources as well as prior knowledge about the cells and tissues to be targeted. Although computational methods have been developed in recent years, they aimed at characterizing the aging process and identifying novel biomarkers [7]. However, one notable exception is a computational method introduced by Janssens et al., which uses machine learning-based classifiers for distinguishing tissue samples from young and old individuals, which are subsequently applied to identify compounds that could rejuvenate cells [8]. Nevertheless, this approach establishes arbitrary thresholds to separate young and old samples and can only inform whether an intervention exceeds these thresholds. Moreover, the definition of young and old individuals influences the results and classifiers typically lose power around the group boundaries. An alternative to classification was introduced in a seminal study that built a machine learning-based method (aging clock) for measuring the age of human tissues and cell types from the methylation state of 353 CpG sites across the human genome [9]. Since then, the concept of measuring cellular age from molecular features has been generalized and led to the development of multiple predictors that exploit different modalities, such as chromatin accessibility, proteomics or transcriptomics [10]. Nevertheless, their utility was limited to serve as biomarkers of aging. However, a pioneering study demonstrated recently the use of a transcriptional aging clock as a readout of a genetic perturbation screen and identified SRSF1 as a novel rejuvenation strategy in human cells and animal models [11].

In this study, we generalize the concept of using a transcriptional aging clock for identifying novel rejuvenation intervention candidates and developed REVIVE, the first computational framework for predicting chemical and genetic perturbations able to restore a youthful transcriptional state in human cells. REVIVE relies on transcriptional profiles of treated and untreated cell types to determine significant changes of their age. To achieve that, we built on a previously introduced transcriptional multi-tissue clock that measures the age of cells and provides insights into the processes declining throughout aging [12]. Moreover, REVIVE ensures that cells revert the ageing hallmarks while maintaining their identity upon perturbation. To demonstrate the utility of our framework, we manually curated a dataset of more than 150,000 human transcriptional profiles of cells treated with more than 10000 perturbagens across different cell types and performed an exploratory analysis to discover perturbations with rejuvenating effects. Indeed, we found that REVIVE recapitulates a significant amount of known perturbations with a lifespan extending effect. Importantly, REVIVE also identifies 477 novel candidate rejuvenating compounds that affect different aging hallmarks. Finally, we demonstrate the utility of our framework to repurpose perturbations that are able to restore youthful transcriptional states for reverting aging phenotypes of different cell types. In summary, we expect this computational framework for predicting chemical and genetic perturbations that can restore a youthful transcriptional state to facilitate the design of new rejuvenation strategies. In this regard, our study provides a novel set of candidates rejuvenating perturbations that prompts the validation in additional human cell types and preclinical animal models.

## RESULTS

### Development of a framework to assess the rejuvenating effect of perturbations

**Figure 1:**
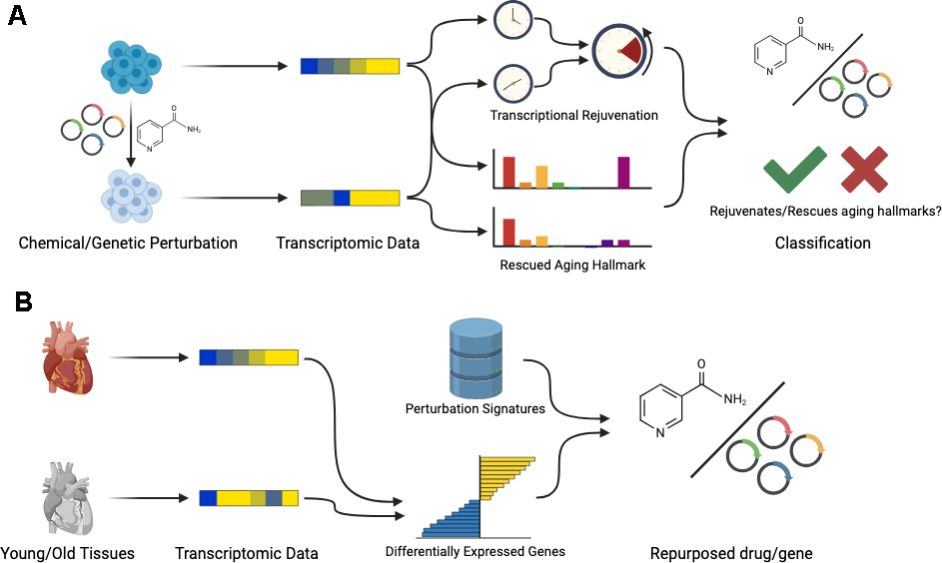
**Workflow of REVIVE.** REVIVE has two different modes: (**A**) Based on a set of transcriptomic samples from perturbation experiments and corresponding control samples, REVIVE estimates the change in the predicted age and the enrichment of aging hallmarks in both groups. Perturbations that show a significant decrease in the predicted age and that rescue at least one aging hallmark are considered to be able to restore a more youthful transcriptional state. (**B**) In order to repurpose perturbations to restore youthful transcriptional states, REVIVE requires a set of differentially expressed genes between a young and old cell type or tissue sample and ranks perturbations based on their potential to revert the expression of the observed differentially expressed genes.

In this work, we propose REVIVE, a computational framework for discovering chemical and genetic perturbations that have rejuvenating effects. REVIVE can be employed in two different ways, namely to detect rejuvenating effects of a given perturbation or to repurpose perturbations with known rejuvenating effects for reverting a transcriptional aging phenotype (Figure 1A, 1B).

**Figure 2:**
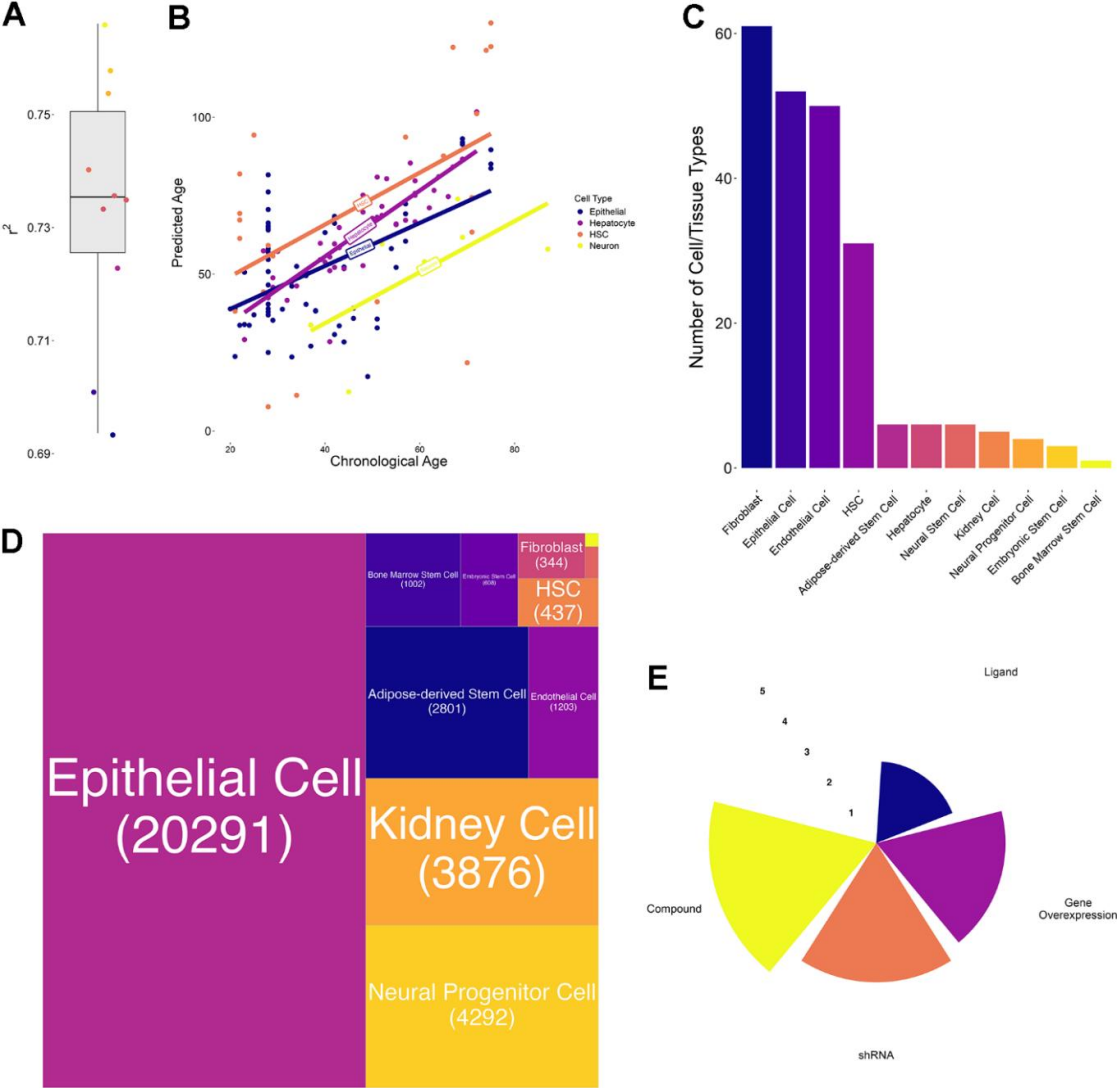
**Transcriptional aging clock validation and characteristics of collected transcriptomic perturbation data.** (**A**) Boxplot of the 10-fold cross-validation performed during training the transcriptional aging clock. The solid lines represent the median and whiskers extend to +/- 1.5*IQR (interquartile range) (**B**) Predicted versus chronological age of independent validation samples in four different cell types: Hematopoietic stem cells (HSC, orange), hepatocytes (purple), epithelial cells (blue) and neurons (yellow). Solid lines represent the regression lines across all points of the same cell type. (**C**) Bar chart depicting the number of cell annotations matching each homogenized cell or tissue type. These annotations subsume different cell lines as well as orthographic representations of the same cell/tissue type. We observed the highest number of annotations for Fibroblasts, Epithelial and Endothelial cells whereas bone marrow stem cells annotations were a priori unique. (**D**) Treemap of the number of perturbations performed in each cell or tissue type. More than 50% of the collected experiments have been performed in epithelial, followed by kidney, neural progenitor, adipose-derived stem and endothelial cells. In total, more than 80% of experiments have been performed in these 5 cell/tissue types. (**E**) Radar plot represents the number of experiments per perturbation type. Values are on a log10-scale.

Specifically, in the first use-case, REVIVE relies on transcriptomic profiles of treated and untreated samples and employs a transcriptional aging clock to quantify the age of both samples. In brief, we built a transcriptional aging clock based on a Generalized Linear Modeling framework with lasso regularization similar to a previously published method [12]. As a training dataset, we employed a previously collected set of RNA-seq samples with available donor age information and restricted it to genes that have been found to be differentially expressed between young (20-29 years old) and old (60-79 years old) donors in at least one tissue in the Genotype-Tissue Expression cohort [12, 13]. Moreover, we removed samples from donors that are younger than 20 years and that have less than 10 million counts resulting in 1350 high-quality observations across 41 different cell or tissue types from diverse organs such as the colon, brain and liver (Supplementary Table 1). The raw RNA-seq counts were subsequently log-normalized and frozen surrogate variable analysis (FSVA) was applied to identify covariates that are not related to chronological age [14]. Automated detection revealed 45 hidden sources of variation, which have been removed from the training dataset. Importantly, processing the data with FSVA allows to overcome a widespread issue with aging clocks, namely the generalizability to new samples without re-training them. For instance, a previous study demonstrated that epigenetic clocks suffer from this issue and significantly benefit from joint normalization [15]. Similarly, transcriptional clocks have been observed to be affected as well, since batch effects produced the strongest signals in a large aging dataset such that their correction was deemed essential [16]. The processed data was then used to train the Generalized Linear Model and thereby select a subset of genes that are most informative about donor age. As a result, a total of 668 genes were selected (Supplementary Table 2 and Supplementary Figure 1). These genes belong to various Gene Ontology terms including general and cell/tissue specific cellular processes (Supplementary Table 3). In particular, the process with the highest number of contributing genes is “Regulation of Cell Migration” followed by “Nervous System Development”, “Positive Regulation of Cell Population Proliferation” and “Negative Regulation of Apoptotic Process”, although no term is significantly enriched (Supplementary Table 3). Interestingly, there is a strong correlation between the number of clock genes in each process and the sum of their importances (Pearson correlation: 0.899). Like for the original clock, we performed 10-fold cross validation on the training data and obtained an increased performance (r-squared: 0.73, range: 0.69 – 0.76) (Figure 2A). To verify our clock on completely independent datasets, we collected RNA-seq profiles of four different cell types with available donor age information and assessed the correlation with the predicted age. As a result, we obtained correlations of 0.52 for hematopoietic stem cells, 0.53 for epithelial cells, 0.66 for neurons and 0.86 for hepatocytes (Figure 2B). Due to the importance of the clock to detect age differences of pro- and anti-aging interventions, we applied our clock to a compiled gold-standard dataset of 80 conditions with known effects. As a result, we found that the direction of the changes in the predicted ages was consistent in 64 out of 80 conditions (binomial test p-value: 5.871e-8), although it needs to be noted that the predicted age differences were small in many cases (Supplementary Table 4).

Finally, REVIVE provides information about the aging hallmarks that are affected by the perturbation as well as a potential loss of cell identity. To achieve that, we obtained sets of genes associated with each aging hallmark from Aging Atlas [17] (Supplementary Table 5) and performed gene set enrichment analysis [18].

To allow for the discovery of known perturbations that can revert a given aging phenotype, we generalized a recently developed approach that relies on differentially expressed transcription factors to identify repurposable drugs [19]. Importantly, due to the fact that aging-related molecular dysregulations do not only manifest in transcription factors, but we also consider the expression of all genes across the genome. In particular, REVIVE employs a single-sample gene set enrichment analysis to quantify the similarity of the observed aging phenotype and the effect of known rejuvenating perturbations.

### Collection of transcriptional chemical and genetic perturbation datasets

In order to apply REVIVE and validate its ability to predict the effect of perturbations to induce a youthful transcriptional state, we obtained publicly available datasets of treated and untreated human samples from LINCS [20]. Importantly, only experiments from non-cancer cells and non-disease samples were considered. Since the experimental data has been derived from the L1000 platform (ligation mediated amplification and capture of its products on fluorescent microspheres), which measures only 978 landmark genes, we obtained pseudo-RNA-seq profiles of all samples from a previously developed generative adversarial network approach [21]. Moreover, we homogenized the sample annotation into 11 cell types and required the presence of appropriate control samples for each perturbation (Figure 2C). As a result, we collected 158,046 transcriptional profiles of cells treated with 10,330 perturbagens. Aggregation of all samples by cell/tissue type, perturbagen, perturbation type, dosage and perturbation time into 34,923 unique groups (Figure 2D). On average, each group contained 4.5 treated (range: 3 – 1175) and 1994.9 control samples (range: 6 – 4104) with the highest groups being epithelial cell control samples. Due to the possibility of multiplexing the experiments, the vast majority of collected perturbations are based on chemicals followed by shRNA-based perturbations and gene overexpression (Figure 2E). Ligand-based experiments, on the contrary, are highly underrepresented (Figure 2E). The complete set of considered samples can be found in Supplementary Table 6.

### Discovery of known and novel perturbations with rejuvenation effects

**Figure 3:**
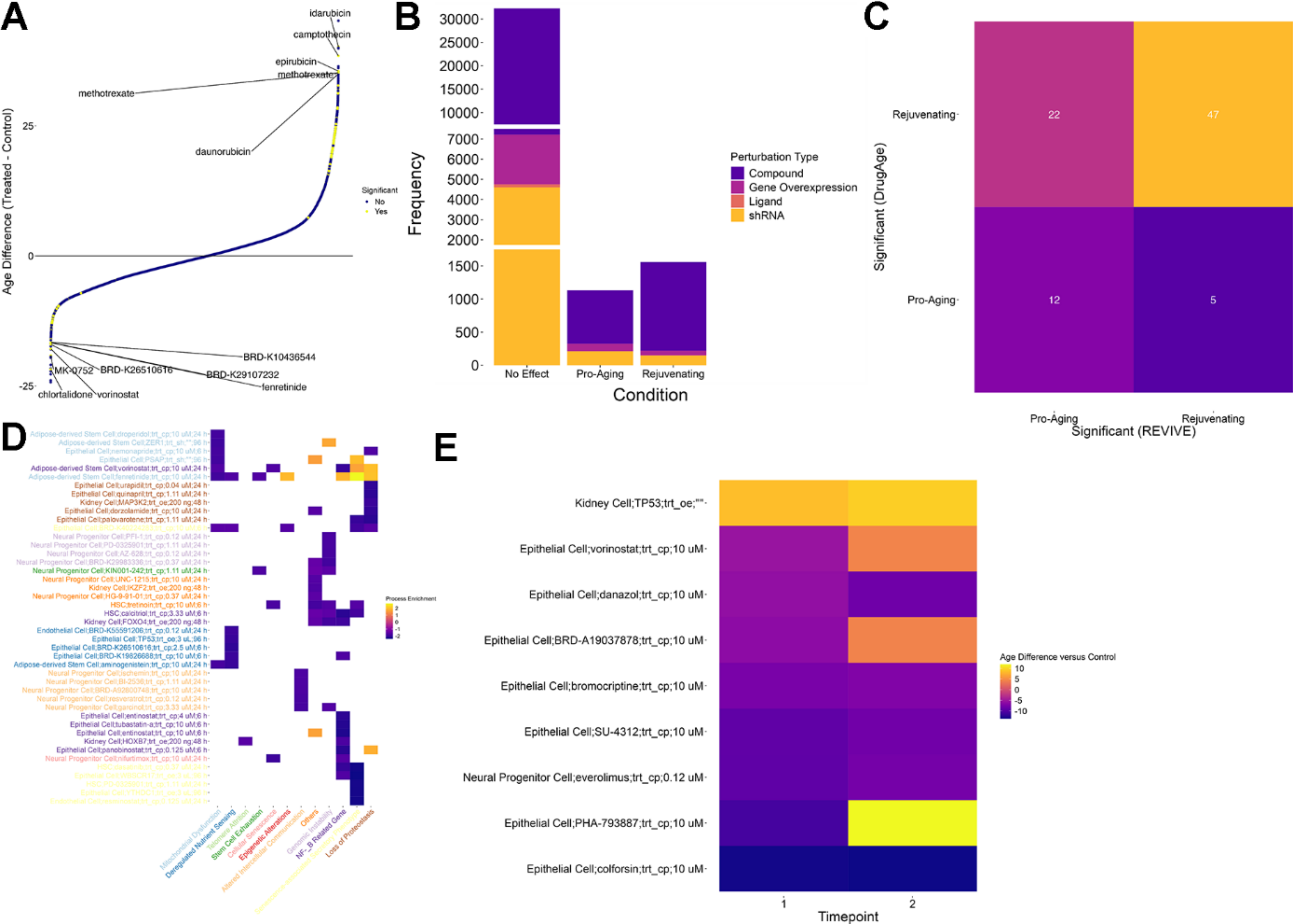
**Screening for perturbations that restore a youthful transcriptional state.** (**A**) Scatter plot of age differences for more than 39000 conditions. Positive values correspond to a pro-aging effect on the transcriptome while negative values signify a more youthful transcriptional state. Significant differences are colored yellow. Significance was determined based on an adjusted p-value of less than 0.01 (t-test, Benjamini-Hochberg correction) (**B**) While most of the tested conditions did not show a significant change in their predicted age, more than 1000 conditions led to a more aged transcriptional state. In contrast, a more youthful transcriptional state could be restored in 1558 conditions. Of these, the vast majority is based on chemical compounds (purple). (**C**) Comparison of compounds that are considered significant in DrugAge (i.e. increasing the lifespan of the treated model organism) with their counterparts in REVIVE. Compounds that showed an adjusted p-value less than 0.01 in any condition were considered significant. The comparison was restricted to compounds (i) that were included in DrugAge and (ii) for which transcriptional samples before and after treatment in any cell type were included in the assembled dataset. Each tile is annotated and colored with the number of cases falling into each category. (**D**) Process enrichment for 12 aging hallmark gene sets for the top 5 effectors of each process among the interventions that restore a more youthful transcriptional state. Negative values (blue) represent the rescuing of a hallmark while positive values (yellow) indicate a pro-aging effect. X- and Y-axis labels are color coded to visualize the processes that are most rejuvenated by each intervention. (**E**) Heatmap of predicted age differences across multiple timepoints for interventions predicted to rejuvenate cells. Higher values (yellow) represent a pro-aging effect whereas lower values (blue) indicate age reversal.

Starting from the collected dataset of chemical and genetic perturbations, we applied REVIVE in an exploratory analysis to all individual transcriptomic profiles and computed the perturbations leading to a reduction of the predicted age (Figure 3A and Supplementary Table 8). The condition was deemed rejuvenating if a t-test resulted in a p-value below 0.01 after multiple testing correction and the mean age difference was negative. As a result, we discovered 1558 conditions that showed a substantial reduction of the predicted cellular age, which corresponds to 742 different perturbations. Importantly, we deliberately distinguish between perturbations and conditions, the latter being defined by the cell type, the perturbation, the perturbation type, and the timepoint after treatment that was measured. Of these, 123 are shRNA-based perturbations, 69 are genetic overexpression, one is ligand-based and 524 are chemical compounds. In contrast, 1132 conditions, corresponding to 655 perturbations, showed a substantially increased cellular age (Figure 3B). Nevertheless, no change was detected for the vast majority of conditions (32233).

To demonstrate the utility of REVIVE, we sought to interrogate whether the effects of perturbations that are supported by experimental studies are correctly recapitulated. In this regard, we obtained experimental data from DrugAge [22] where we consider both counteracting the functional cellular decline as well as lifespan extension in at least one experiment as rejuvenating effects. Compounds for which transcriptional samples of treated and untreated cells are included in our perturbation dataset were retained for comparison. As a result, we found that 47 out of 525 compounds predicted to rejuvenate by REVIVE were supported by DrugAge (Supplementary Table 7). Conversely, 12 out of 336 compounds predicted to be pro-aging showed lifespan-reducing effects in DrugAge (Supplementary Table 7). Although 27 compounds have been shown to possess opposing effects, the predictions are significantly associated with the experimental evidence provided by DrugAge (Fisher test p-value: 0.005, Figure 3C). Thus, we expect REVIVE to produce reliable, testable hypotheses of perturbations that can rejuvenate the transcriptome of cells.

Among these perturbations are well-known compounds, such as alpha-estradiol and resveratrol, which show a decrease of 8.7 and 6.1 years, respectively. Notably, aspirin and metformin did generally not show a significant change in the predicted cellular age upon perturbation. On the contrary, higher doses of aspirin (>10 uM) showed increasing transcriptional ages in epithelial cells in a dose-dependent manner. Moreover, although both drugs demonstrated lifespan extending effects in model organisms including mice, C. elegans and D. melanogaster, the rejuvenating effect on human cells and organs remains elusive [23].

Next, we assessed which conditions could improve the hallmarks of aging by performing gene set enrichment analysis of the log-fold-changes of each condition in curated gene sets for each hallmark [24]. As a result, we found that 329 of 1558 conditions predicted to rejuvenate improve at least one aging hallmark. The top 5 interventions affecting each of the hallmarks are shown in Figure 3D. In this regard, it is important to note that “stem cell exhaustion” is a hallmark that only applies to stem and progenitor cells. Nevertheless, since 9160 out of the more than 34000 perturbations have been conducted in stem/progenitor cells, we consider it important to include it in this assessment as well. For instance, dasatinib applied to HSCs shows significant negative enrichment in “Senescence-associated Secretory Phenotype” and “NF-kB Related Genes”, which is supported by previous studies due to the proven effect of dasatinib+quercitin as a senolytic [25]. Interestingly, of the 329 conditions that improve at least one aging hallmark, 115 alleviate the senescence-associated secretory phenotype while the remaining conditions show no or negative, pro-aging, effects.

The compound that alleviates the most aging hallmarks (five) is BRD-K40224283, a chemical compound whose mode of action is unknown. In particular, the compound showed an age reduction of 7.3 years after 6 hours of treatment in epithelial cells and its effect are enriched in “Senescence-associated Secretory Phenotype”, “Loss of Proteostasis”, “Epigenetic Alterations”, “Mitochondrial Dysfunction” as well as “Deregulated Nutrient Sensing”. Interestingly, the rejuvenating effect of BRD-K40224283 seems stable after 24h (-9.9 years) although no statistical significance was attained.

Finally, we set out to interrogate the time-dependent changes of the rejuvenating or pro-aging effects of perturbations for which multiple timepoints were measured. Although only nine interventions had multiple timepoints of which all showed significant age difference, we made an interesting observation (Figure 3E). In particular, one perturbation, the overexpression of TP53 in Kidney cells, showed a consistent pro-aging effect. In contrast, five compounds resulted in a persisting rejuvenation effect including colforsin (forskolin), everolimus, SU-4312, bromocriptine and danazol. On the contrary, the rejuvenating effect of vorinostat (HDAC inhibitor), BRD-A19037878 (trichostatin A; HDAC inhibitor) and PHA-793887 (CDK inhibitor) on epithelial cells reverses into a pro-aging effect in the second timepoint suggesting that a continuous supply of the drug is needed to sustain its effect. However, it remains elusive whether these observations are due to intrinsic cell type specific differences or are inherent to the mechanisms of action of the drugs.

### Repurposing chemical and genetic perturbations for reverting aging phenotypes

**Figure 4:**
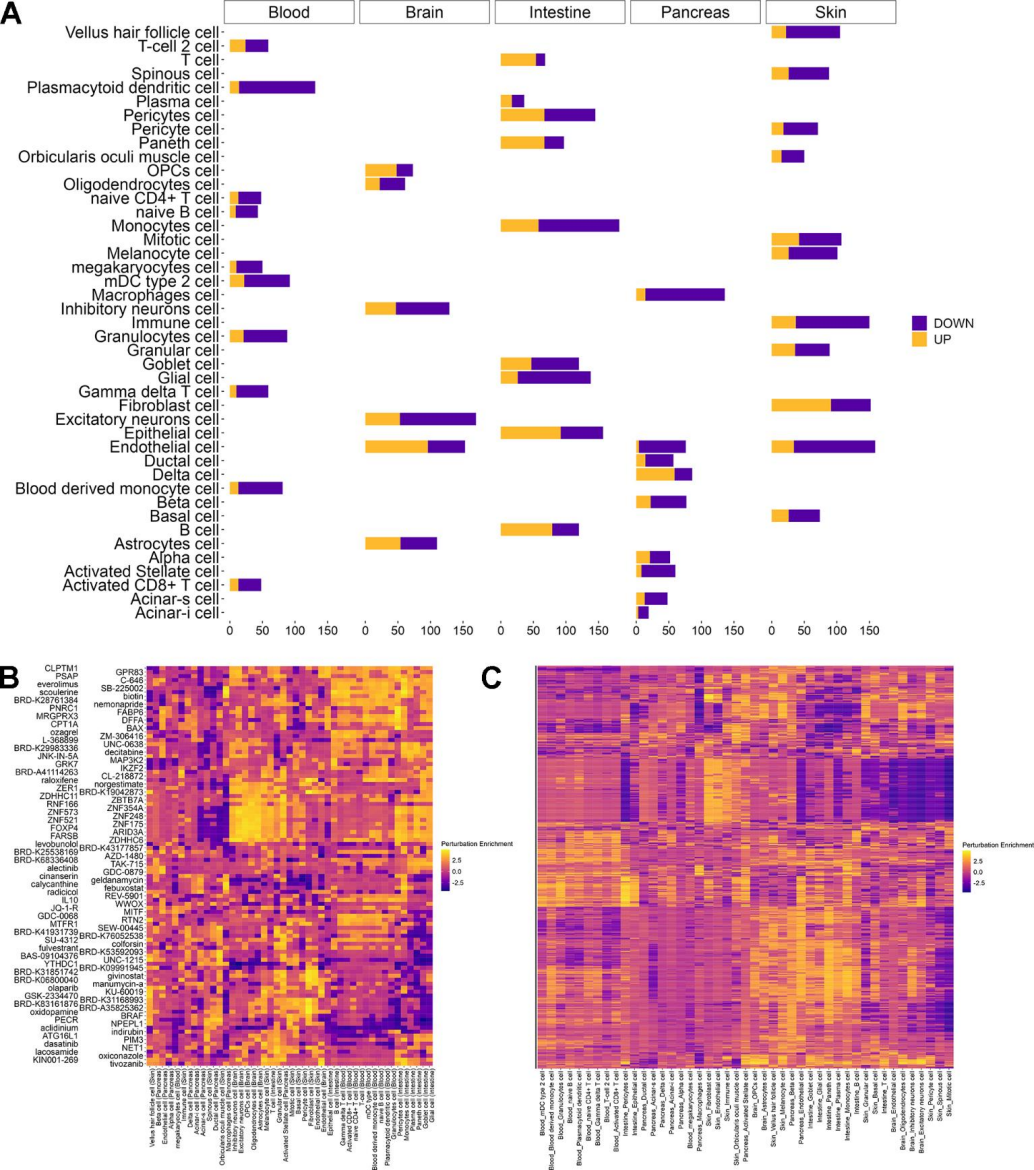
**Repurposing perturbations to revert aging phenotypes.** (**A**) Number of differentially expressed genes between young (0 – 19 years) and old (60 – 100 years) individuals per cell type in each tissue as obtained from AgeAnno. Genes that are upregulated during aging (yellow) are distinguished from those that are downregulated (purple). (**B**) Heatmap of the two interventions having the highest perturbation score in each cell type. Perturbation enrichment is shown across all cell types including those in which the perturbation does not have beneficial effects. Higher scores (yellow) indicate a perturbation to revert a higher fraction of age-related differentially expressed genes whereas negative scores (blue) indicate a reinforcement of the pro-aging changes. (**C**) Heatmap of perturbations scores for all candidates rejuvenating perturbations in 45 cell types from different tissues. Higher scores (yellow) indicate a perturbation to revert a higher fraction of age-related differentially expressed genes whereas negative scores (blue) indicate a reinforcement of the pro-aging changes.

In addition to predicting the effect of chemical and genetic perturbations, REVIVE allows for the repurpose of perturbagens to counteract aging phenotypes, i.e. the transcriptional differences of a cell type in old individuals compared to young ones. In this regard, REVIVE compares the differentially expressed genes between old and young cell types that are related to aging hallmarks with the phenotypes induced by perturbations that were predicted to induce a more youthful transcriptional state (Figure 1B, see methods for details). To support this approach, we obtained differentially expressed genes of 45 cell type and tissue samples between young (0-19 years of age) and old (60-100 years of age) donors from AgeAnno [26] (Figure 4A). After applying REVIVE to obtain the perturbations that resemble the age-related differences best, we observe several cases in which interventions with a demonstrated effect have been predicted (Figure 4B and Supplementary Table 9). For instance, among the top-ranking interventions to induce a younger phenotypic state in immune cells is everolimus. Indeed, mTOR inhibitors, as is everolimus, have been shown to improve immunity in older human beings in clinical trials [27]. Another intervention that showed high enrichment scores in different skin cell types is colforsin (forskolin). Indeed, topical forskolin treatment of the skin has been shown to induce melanization, epidermal cell accumulation and skin thickening [28]. This exemplifies that perturbation signatures exhibit stronger effects than cell type signatures. This is further supported by clustering the cell types by their prediction score for each perturbation (Figure 4C). Indeed, cells of the same type show similar enrichment patterns for perturbations, which highlights the validity of our approach. Thus, we expect it to be generalized to cell types in which no experiments have been conducted.

## DISCUSSION

In this study, we propose REVIVE, the first computational framework for predicting chemical and genetic perturbations in human cells that restore a youthful transcriptional state. Previously introduced computational approaches that have been developed in the context of drug repurposing for diseases relying on genome-wide comparisons [29] often fail in predicting the rejuvenating effects of perturbagens since they are overshadowed by other, unrelated effects of perturbations. For instance, rapamycin exerts its rejuvenating effect through inhibition of mTORC1, but concomitantly induces changes in glucose and lipid metabolism mediated by mTORC2 as well as in the proliferation rate [30]. In order to circumvent this issue, REVIVE solely focuses on age-related genes and uses a transcriptional aging clock that is predictive of the age of the samples. In addition, most of these previously introduced tools rely on transcriptional perturbation signatures that have been largely derived from cancer cells, which are known to exhibit various alterations at the signaling and transcriptional level [19]. In that regard, REVIVE relies on a dataset of non-cancer cells to identify transcriptional perturbation signatures. Indeed, a recent approach supports the use of non-cancer cells to improve drug repurpose based on the expression of transcription factors [19]. However, the expression of only a few transcription factors can be associated with aging, rendering them unable to inform about potential rejuvenation effects [31]. Indeed, of the genes that REVIVE selected to be predictive of age, only a few are transcription factors.

Despite known perturbations, REVIVE discovered 742 perturbations that were able to restore a youthful transcriptional state. Interestingly, while some of these perturbations exerted an effect on multiple cell types, the majority only restored a youthful transcriptional state in a single condition. Indeed, cell type specific effects have been observed before, such as in the case of different brain cell types [32]. In particular, cell type specificity is also observed for perturbations that affect general pathways, such as GDC-0068 that targets Akt/PI3k-signaling. On a different note, we demonstrated that REVIVE can be used to repurpose drugs for inducing youthful transcriptional states in any cell type. Although previous studies explored the possibility of employing general purpose drug prediction tools in the context of diseases, such as L1000CDS2 [29], the success of these approaches has been limited. We hypothesize that the effects of drugs on age-related transcriptional changes are overshadowed by other drug effects. In contrast, REVIVE specifically focuses on changes related to genes associated with cellular age, which allowed us to predict known as well as novel perturbagens for restoring youthful transcriptional states.

One interesting aspect when using transcriptional aging clocks as a surrogate for discovering rejuvenating perturbations is that the r-squared of the aging clock does not need to be high. Instead, a weaker condition is sufficient to perform this task, namely that the ranks of the age differences are concordant. This is evidenced by the fact that different aging clocks lead to vastly different age predictions but can recapitulate the effect of known pro- or anti-aging interventions, which is sufficient for the purpose of discovering novel rejuvenating perturbations [12]. For instance, the epigenetic clock introduced by Horvath [9] obtains higher r-squared values on testing sets with respect to chronological age predictions compared to our transcriptional clock (0.92 versus 0.73), however, it is still unable to capture the effects of various interventions that are known to increase the lifespan of cellular populations [33]. In contrast, the transcriptional aging clock we employ in this study was shown to capture various different interventions with known rejuvenating or pro-aging effects. Moreover, it is especially important when considering the application of REVIVE to cell types that were not included in the training data and provides a more robust method design compared to the reliance on the actual ages.

Despite the demonstrated ability of REVIVE to discover rejuvenating perturbations, it bears some limitations. Namely, aging, and therefore rejuvenation, is reflected in different molecular modalities, such as epigenetics, metabolomics, transcriptomics and proteomics, at varying degrees. Thus, it is likely that REVIVE cannot detect all chemical and genetic perturbations, but only those whose rejuvenation effect is mediated by transcriptional changes. Similarly, REVIVE employs a multi-tissue aging clock for assessing the restoration of youthful transcriptional states by perturbations. Although the genes it considers are reflective of age, specialized processes of cells and tissues are neglected. For instance, a recent study developed a fibroblast-specific aging predictor based on eight cellular processes which vastly different to the processes enriched in our multi-tissue clock genes [11]. Taking together, this suggests that cell type and tissue specific anti-aging effects cannot fully be captured. However, due to the generality of our proposed framework, we hypothesize that the incorporation of tissue or cell type specific aging clocks would lead to the discovery of additional perturbagens that are able to restore a youthful transcriptional state.

In summary, REVIVE provides the first computational framework for systematically identifying perturbagens in human cells that restore youthful transcriptional states based on transcriptomic profiles. Thus, it paves the way for the evaluation of large-scale genetic and chemical screens and is therefore expected to accelerate the design of novel rejuvenation strategies.

## MATERIALS AND METHODS

### Preprocessing of transcriptomics data

The transcriptomics data collected for this study was generated with ligation-mediated amplification followed by capture of the amplification products on fluorescently addressed microspheres (L1000), which were initially preprocessed independently. In particular, the latter technology was employed to create the LINCS resource and relies on the measurement of 978 “landmark” genes that can be used to infer the remaining transcriptome [20]. Although Subramanian et al. provides a methodology for inferring the expression of 11350 genes, we employed a recently published methodology to convert L1000 generated data to pseudo-RNA-seq samples. Using a generative adversarial network approach, this method allowed the inference of 23614 genes from the originally measured 978 [21]. Taking this approach enables the deconvolution of age information contained in the originally measured genes and thereby increasing the predictive power. To confirm that the approach produces reasonable pseudo-RNAseq samples, we collected RNAseq samples for 22 cell types contained in LINCS performed pairwise Pearson correlation analysis. As a result, the median correlation we observed was in all cases above 0.75 although some cell types, such as A549, HEK293T or A375, showed a considerable number of outliers (Supplementary Figure 2). In this regard, we employed the publicly available workflow of the method with default parameters.

For training our transcriptional clock, we obtained the data that was used for training MultiTIMER [12]. RNA-seq reads were downloaded from the Sequence Read Archive and aligned with kallisto v0.50.1 [38]. A transcriptome index was built using the kallisto index subprogram with default parameters on the FASTA sequences from cDNA and non-coding RNA from Ensembl 111. Transcript isoform expression was estimated using the kallisto quant subprogram. In case of single-strand data, the fragment length was set to 200 bp while strand-specificity of paired-end reads was determined by randomly sampling 50000 reads and aligning them to the transcriptome.

Due to the fact that the number of measured genes varies between samples profiled with different technologies, we selected those that are in common. Training samples that had less than 10 million counts were a priori excluded.

### Predicting the effect of perturbations on sample age

To assess whether perturbations can restore a youthful transcriptional state, we a transcriptomic clock. Since we used a subset of the training data that was used for MultiTIMER [12], we also obtained the corresponding chronological age information for these samples from the original publication. For pre-processing, raw counts were log-transformed and surrogate variable analysis (sva) was on the training data [34]. Automatic estimation of the number of surrogate variables yielded 45 hidden covariates and removed them using the frozen sva approach [14]. The training data was subsequently subset to genes that are differentially expressed between young (20-29) and old (60-69 or 70-79) donors in at least one tissue from the Genotype-Tissue Expression project [13]. Finally, a generalized linear model was trained with lasso regression using the h2o R package [35]. In particular, the IRLSM solver was employed with automatic lambda estimation and automated data standardization. Training weights for individual samples were computed to balance differences in cell type and age representations as the inverse of the cell type or age frequencies. After training, the final model was applied to all intervention data. Next, we subtracted the average age of the treatment samples from the age of the corresponding control profile in order to obtain the average age difference. Here, negative values correspond to a more youthful transcriptional state while positive values signify a pro-aging effect. A t-test has been employed to assess significance and multiple testing correction (Benjamini-Hochberg) has been applied to adjust the resulting p-values. Conditions with an adjusted p-value less than 0.01 were deemed to have a significant rejuvenating (in case the age difference is negative) or pro-aging (in case the age difference is positive) effect.

### Determining changes in the hallmarks of aging after perturbation

In order to determine whether perturbations not only restore a younger age based on a transcriptional clock but also revert the expression of genes related to known dysregulated biological processes, we obtained a collection of 416 genes and their association to aging hallmarks from the Aging Atlas [17]. Next, we performed Gene Set Enrichment Analysis implemented in the ‘fgsea’ R-package on the log-fold changes between treatment and corresponding control sample. Negative normalized enrichment scores correspond to an alleviation of the hallmark whereas positive scores signify a deterioration.

### Predicting repurposable drugs for aging phenotypes

We pre-computed the average log fold change for all interventions by computing the average expression across all control and treatment samples, respectively, and subtracted the log-transformed values in such a way that positive values signify an increased expression in treatment samples whereas negative values correspond to a higher expression in control samples. Given a list of differentially expressed genes, we create two sets corresponding to up- and down- regulated genes, respectively. Using the log2 fold changes of each intervention that was predicted to restore a youthful transcriptional state, gene set enrichment analysis is performed against up- and down-regulated differentially expressed genes using the fgsea R package with default parameters [36]. Finally, a prediction score is computed by subtracting the score for the upregulated from the score for the downregulated gene set.

### Data availability

All accession numbers to datasets used within this study can be found in Supplementary Table 4.

### Code availability

The custom R code created for this study is available at: https://github.com/saschajung/REVIVE

## Supplementary Material

Supplementary Figures

Supplementary Table 1

Supplementary Table 2

Supplementary Table 3

Supplementary Table 4

Supplementary Table 5

Supplementary Table 6

Supplementary Table 7

Supplementary Table 8

Supplementary Table 9
