# Supplementary material for: REVIVE: a computational platform for systematically identifying rejuvenating chemical and genetic perturbations
Source: Aging (Albany NY). 2025 Nov 25;17(11):2844–58. doi: 10.18632/aging.206342 (PMC12705183; doi:10.18632/aging.206342)
Supplement: Supplementary Figures [file aging-17-11-206342-s001.pdf]

SUPPLEMENTARY FIGURES

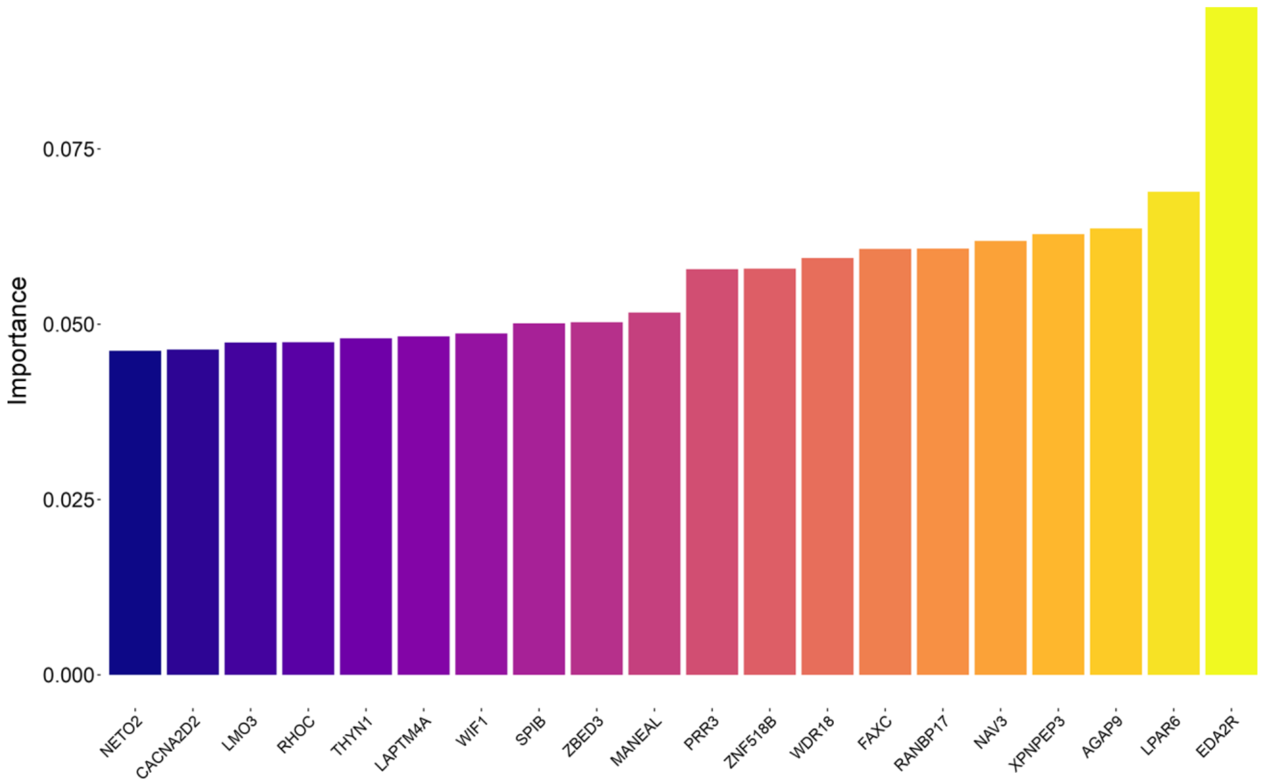

Supplementary Figure 1. Bar chart of the predictive genes in the transcriptional clock with the highest importance.

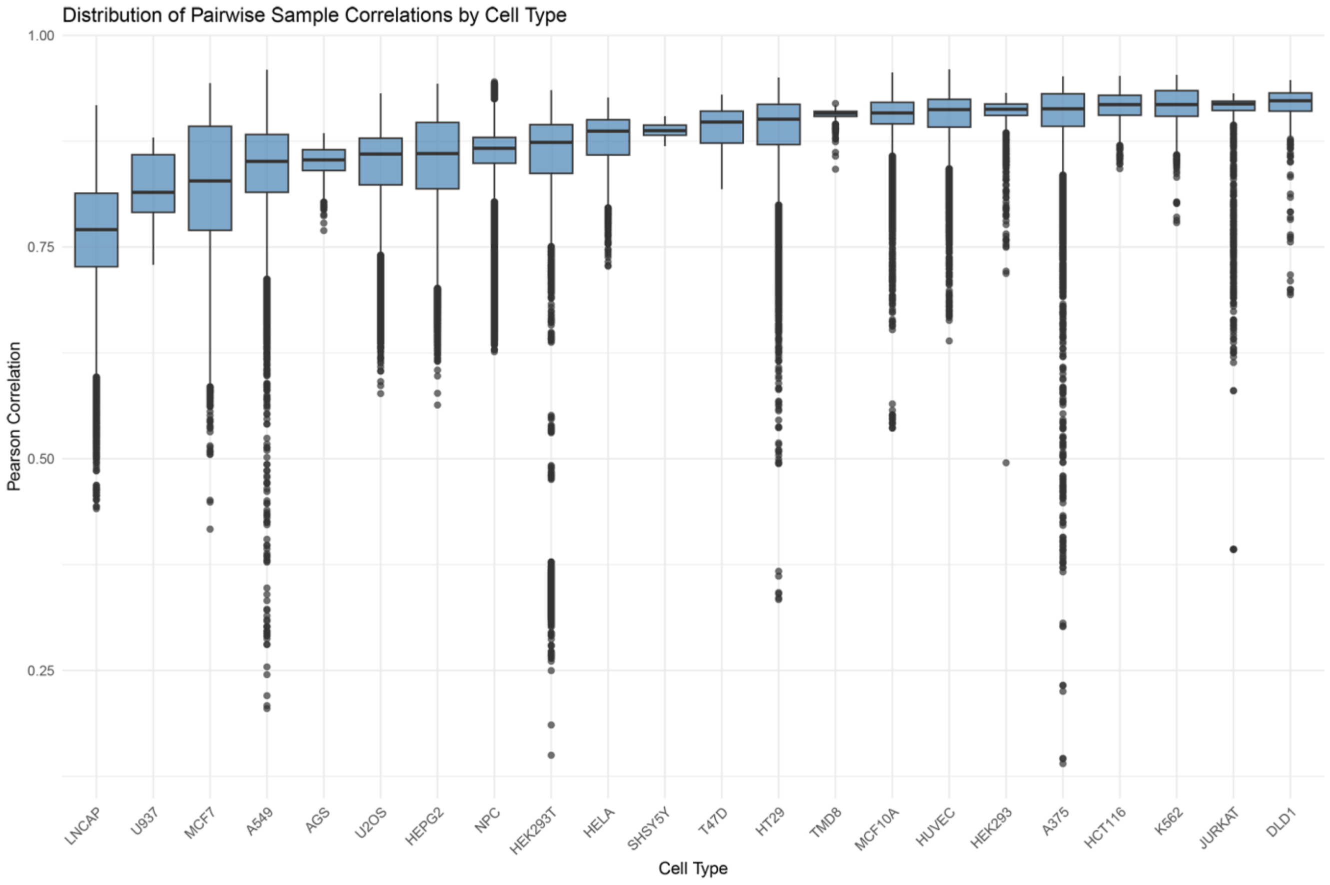

Supplementary Figure 2. Boxplots of pairwise Pearson correlation coefficients between RNAseq samples and pseudo-RNAseq samples generated from untreated cell lines contained in the LINCS database.
